# Could the Supertowel be used as an alternative hand cleaning product for emergencies? An acceptability and feasibility study in a refugee camp in Ethiopia

**DOI:** 10.1371/journal.pone.0216237

**Published:** 2019-05-06

**Authors:** Sian White, Jessica F. Petz, Kifle Desta, Torben Holm Larsen

**Affiliations:** 1 Department of Disease Control, London School of Hygiene and Tropical Medicine, London, United Kingdom; 2 Independent Consultant, Tigray, Ethiopia; 3 Real Relief ApS, Kolding, Denmark; University of South Florida, UNITED STATES

## Abstract

**Background:**

Diarrhoeal diseases are a major contributor to morbidity and mortality in humanitarian crises. Handwashing with soap may reduce diarrhoea by up to 47%, however, the circumstances associated with displacement make it challenging for crisis-affected populations to be able to wash their hands with soap. The Supertowel is an alternative hand-cleaning product, proven to be as efficacious as handwashing with soap. The Supertowel is a micro-fibre towel with an anti-microbial treatment. When dipped in water it is capable of removing and killing pathogens from hands. This study aims to assess whether the Supertowel could be an acceptable and feasible product for crisis-affected populations.

**Methods:**

The study took place in an Eritrean refugee camp located in Tigray state in Ethiopia. We used a mix of qualitative methods to understand use and acceptability, including baseline observations (n = 13), behaviour trials involving interviews at three time points (n = 19) and focus group discussions (n = 3). We thematically analysed data from interviews and discussions.

**Results:**

Participants indicated that the Supertowel was convenient, easy to use and saved them water and money. All households participating in the behaviour trials had at least one Supertowel in use at the end of the trials (follow-up visit two). In discussions participants reported that the Supertowel was more desirable than comparable hand cleaning products. In interviews, trial participants explained that the product enabled them to clean their hands at times when they might not normally bother. The research also identified some issues with the smell of the Supertowel and its intuitive use.

**Conclusions:**

The Supertowel was found to be an acceptable and useful hand-cleaning product that could complement soap use in crisis contexts. This pilot study also identified areas of future research including the need to compare different distribution models for the Supertowel (distribution in hygiene kits compared to distribution with an accompanying communication package) and to evaluate its use at scale over a longer time period.

## Introduction

Humanitarian crises often displace populations and relocate them into crowded environments. During crises it is also common for water and sanitation infrastructure to be damaged and health systems to be weakened or overburdened. These conditions are ideal precursors for the spread of pathogens through the faecal-oral route. It is estimated that in camp situations, 40% of all mortality during the acute phase of a crisis is due to diarrhoeal disease [1]. Evidence from non-emergency settings indicates that handwashing with soap may reduce diarrhoea by 23–47% [2, 3]. However, in emergencies water is typically scarce and distribution of soap is complex and has had mixed results on improving handwashing behaviour [4–6]. Where soap and water are available in crises contexts they are typically not conveniently available for handwashing and are often prioritised for other household tasks (e.g. bathing, laundry and dishwashing) [5, 7]. Recent literature reviews have cited the many challenges and limitations of hygiene promotion in emergency settings [8, 9]. This study explores whether an alternative hand-cleaning product could be acceptable and feasible for crisis-affected populations.

### The Supertowel

The Supertowel is a hand-cleaning product, designed specifically for emergency settings. The Supertowel uses a dual mechanism to remove pathogens from hands and subsequently kill them. Firstly, its microfiber composition allows the Supertowel to remove 99% of pathogens from hands [10]. In order to achieve this, the Supertowel must be dipped into water and then rubbed over the hands and fingers. The unique feature of the Supertowel is its second mechanism of action–a permanent anti-microbial bonding. Once pathogens are transferred to the Supertowel they are attracted to chains of carbon atoms. These are attached to positively charged nitrogen atoms which are bonded to a silica layer of the fabric. The positively charged layer attracts negatively charged microbes causing membrane disruption and death. The anti-microbial technology does not involve toxic chemicals and the efficacy of the treatment does not diminish over time. The technology has been previously applied to hospital linen and reusable menstrual hygiene pads [11–15] and can effectively kill 99% of bacteria, protozoa, fungi and encapsulated viruses in 30 seconds (*tests performed on escherichia coli*, *staphylococcus*, *aspergillus brasiliensis*, *aspergillus niger*, *pseudomonas aeruginosa*, *clostridium sporogenes*, *klebsiella pneumoniae*, *methicillin resistant staphylococcus aureus and of candida albicans*). In a laboratory–based efficacy study we demonstrated that the Supertowel was more efficacious at removing non-pathogenic *E*.*coli* from pre-contaminated hands than handwashing with soap (Mean log_10_- reduction of 4.11±0.47 for ST1, vs 3.01±0.63 for soap, (p<0.001)) [16]. The same study showed that the Supertowel required much less water than handwashing with soap (less than 80ml compared to 1.2L for handwashing with soap) under controlled conditions and was considered preferable in communal settings. The SuperTowel has several other potential benefits in emergencies. Unlike soap it will not need to be frequently distributed as the antimicrobial treatment does not deteriorate with time [12]. It is smaller and lighter than soap making distribution easier and it is likely to be more cost-effective with the estimated cost per towel being 50 cents.

## Objectives

Having proved the efficacy of the Supertowel in controlled conditions, this study aimed to assess the acceptability and feasibility of the product in a humanitarian context where water is scarce and ongoing soap distribution is challenging. The study also aimed to provide opportunities for crisis-affected populations to generate ideas about how the product could be improved to better meet their needs.

## Methods

### Study setting

The study took place in Hitsats Camp, an Eritrean refugee camp located in Tigray state, Northern Ethiopia. This research was conducted in September 2018. At this time the camp population was approximately 12,000. A large proportion of the refugees in Hitsats Camp were young males fleeing military conscription in their home country of Eritrea [17]. Families were often unable to flee Eritrea as a unit. Within the camp it was common to find ‘combined families’ where members of different families are assigned to one shelter; ‘foster households’ where families have adopted children who arrived at the camp without their guardians; and ‘single-person households’ where unmarried members of the same sex reside in the same shelter. Hitsats camp was chosen as the site for this research because its refugee residents face major water challenges both in terms of the quantity available, and the consistency of the supply. For the majority of the year refugees have access to 7 litres of water per person per day, from boreholes. This research was conducted during the rainy season, when water access typically increases to 20 litres of water per person per day. The population of the camp receive soap distributions on a monthly basis (one bar of soap per person) and have to purchase any additional soap they require from markets within the camp. Hygiene promotion was being conducted on an ongoing basis by trained camp-based volunteers who conducted household hygiene visits. Sixty handwashing facilities had been installed in the camp, but none were observed to be in a functional state at the time of this research.

### Baseline observations

The research team used a form of maximum variation purposive sampling [18] to select households to participate in the unstructured baseline observations. Household selection was based on a transect walk through each of the camp regions with social workers. Through discussion we tried to include different types of households, different religious and ethnic groups, and people with different durations of residence in the camp. Observations took place in the mornings between 8am and 11am to capture key hygiene related events including handwashing at critical times (handwashing after using the toilet, after cleaning a child’s bottom, before food preparation, before eating and before feeding a child). Participants were not explicitly told that we were studying handwashing behaviour, rather they were informed that we were there to learn about normal daily routines in the camp. The aim of the unstructured observations was to understand current hand-cleaning practices, the factors that facilitate and constrain this, and to generate a broad understanding of the camp context.

### Behaviour trials

Following the observations, the research team used the same purposive process to select households for the behaviour trials. Behaviour trials were used to understand user perceptions of the Supertoweland barriers to use. Behaviour trials have been used to assess handwashing behaviour in several studies [19, 20]. The behaviour trials took place over 13 days and consisted of three interactions with each family (there are referred to as ‘the initial visit’, ‘follow-up visit one’ and ‘follow up visit two’). During the initial visit the research team explained the Supertowel to all members of the household and demonstrated how to use it for hand cleaning (based on the WHO steps for hand cleaning[21]), before conducting a short, recorded interview to get the family’s initial impressions about the product. During this visit, participants were reminded that handwashing with soap is an effective way to clean their hands but that we would like them to try using the Supertowel for handwashing or to supplement handwashing with soap for the duration of the trial. Five days later, the research team returned to conduct the follow-up visit one. During this visit we collected information about the socio-demographics of the family; recorded where each Supertowel was being kept; assessed whether each Supertowel was wet and whether it smelled; and then conducted a longer recorded interview with the family about their experiences using the product, including any challenges they had experienced. At this time point participants were also invited to try using the SuperTowel for purposes other than handwashing if they wished. Follow-up visit two was conducted 12 days after distribution of the Supertowel. During this last visit to each family the location, we recorded the wetness and smelliness of each of the Supertowel and interviewed family members about their ongoing experiences of use and the likelihood that they would continue to use the Supertowel after the conclusion of the research. The behaviour trial guides are included as part of the supplementary materials (S1 Guide).

At the initial visit we told participants of the duration of the trial and that we would return several times, but they were not told the exact day we would visit, in order to reduce bias. Measuring the wetness was designed to be a proxy indicator of use given that the Supertowel can only be used effectively for hand-cleaning if it is wet or damp. Literature around handwashing indicates that pleasant smells encourage people to wash their hands [22] and therefore it was assumed that if the Supertowel had an unpleasant odour it may discourage use. Due to the subjectivity of this type of assessment, both indicators were assessed on a scale of 1 to 4 by one of the authors (THL) in order to maintain consistency of grading. The wetness scale classed a Supertowel that was wet as 4, damp as 3, almost dry as 2, and dry as 1. The smell scale classed a Supertowel with no smell or a pleasant odour as 4, a slight smell as 3, smelly as 2, and very smelly as 1. The Supertowel used in this study were produced by Real Relief and were distributed to participants in a waterproof fabric bag with a pull string. The carrier bag was designed to allow users to take the Supertowel with them when travelling outside the home.

### Focus group discussions

Once the behaviour trials had concluded, the research team conducted three focus group discussions using two different formats. The first format was designed to understand perceptions about the Supertowel in comparison to other hand-cleaning products. This format was conducted with a group of seven women and a separate group of seven men. In these two focus group discussions we introduced participants to a range of hand-cleaning products (bar soaps, liquid soap, alcohol hand rub, and the Supertowel). Participants were then encouraged to try each cleaning product in a random order and to reflect on how their hands felt afterwards and what they liked or disliked about each product. Participants were then asked to collectively rank the products according to a range of criteria. The second type of focus group discussion was designed to understand what types of instructions should accompany the Supertowel and the mode through which these messages should be delivered. This focus group discussion was conducted with a group of seven women who were shown the Supertowel (without any explanation) and a series of pictograms about how to use it. We then asked them to demonstrate how they would use the product given the image-based instructions. Through open discussion we asked the women to brainstorm alternative ideas for explaining how the Supertowel should be used and how it could be made more intuitive. Social workers from the camp helped the research team to purposively select participants for these focus groups. Participants were selected to include different regions of the camp and to reflect different ages, literacy levels, durations of displacement and religions. The focus group discusion guides are included as part of the supplementary materials (S2 Guide).

### Data collection and management

The data collection team was comprised of six people; one researcher associated with a UK research institute (SW); one European product expert from the organisation which designed the Supertowel, Real Relief (THL); one local project manager from the Danish Refugee Council (DRC), one local translator; and two camp residents who were volunteer social workers for DRC. The team received a one-day training on the research methods (facilitated by SW) and ongoing guidance throughout the data collection to minimise errors.

Baseline observational data was collected using free form notes. Data collectors were instructed to document any activity in the household and the time at which it occurred, regardless of whether the activities were related to the target behaviour (hand-cleaning) or not. Data from observations were entered into Excel each evening to avoid any data loss. All the focus group discussions and the interviews that occurred during the behaviour trials were audio recorded, translated and transcribed. Scaling of the wetness, smell and location of each Supertowel was recorded by hand, and then entered into Excel at the end of each day.

The study was designed to adhere to the COREQ criteria for reporting qualitative research [23] (see S1 Table).

### Data analysis

Baseline observational data were coded to highlight handwashing events, or critical times for handwashing that were missed, as previously described. Hand-rinsing with water alone and handwashing with soap were categorized separately. Transcripts from all behaviour trial interviews and focus group discussions underwent a thematic analysis using the question guides as the initial analysis framework and then identifying emerging themes. Two researchers (SW and JP) conducted the thematic analyses. Scaling data related to wetness and smell was analysed descriptively at both household and individual levels. Use of the Supertowel was assumed if a towel was ranked as a 3 or 4 on the wetness scale.

### Consent, assent and ethics

Informed written consent was obtained from all study participants over the age of 18. Information about the study was provided in writing in Tigrinya but was explained verbally by the third author (KF) to all participants to ensure understanding. Where participants were unable to sign their name, a thumbprint was used to indicate consent. Informed assent was also obtained for all household members aged 7–16. For minors who were under the age of 18 and living independently in the camp, informed written consent was provided by the camp management and by the individual. Ethical approval for this study was granted by the London School of Hygiene and Tropical Medicine. Local permission was provided by the Administration for Refugee & Returnee Affairs.

## Results

After conducting baseline observations in 13 households the research team concluded that a degree of saturation had been reached in relation to understanding current hand-cleaning practices. All of these 13 households agreed to participate in the behaviour trials as well. A further six households were sampled to participate in the behaviour trials only, for a total of 19 households. Table 1 summarises the socio-demographic characteristics of all households, and individuals. Eighteen households perceived both water accessibility and soap availability and affordability to be major issues in the camp. However, soap of some kind was present in all 19 households.

**Table 1:** Socio Demographics of Eritrean refugees in Hitsats Camp who participated in the observations and/or behaviour trials.

**Household Demographics N = 19**	
Household Size	
*1–4 people*	*4 (21%)*
*5–9 people*	*12 (63%)*
*10+ people*	*3 (16%)*
Family unit	
Yes	*13 (68%)*
No	*6 (32%)*
Soap Present	
Yes	19 (100%)
No	0 (0%)
Is water access a challenge for your household?	
Yes	18 (95%)
No	1 (5%)
Is soap access and affordability a challenge for your household?	
Yes	18 (95%)
No	1 (5%)
**Individual Demographics N = 128**	
Age	
*0–10*	*39 (30%)*
*11–17*	*23 (18%)*
*18–30*	*44 (34%)*
*30+*	*20 (16%)*
Gender	
Male	*67 (52%)*
Female	*61 (48%)*
Religion	
Christian	*114 (89%)*
Muslim	*14 (11%)*
Duration in camp	
1–5 months	*21 (16%)*
6–11 months	*13 (10%)*
12–23 months	*50 (39%)*
24+ months (max duration 5 years)	*42 (33%)*

In total, 84 critical opportunities for handwashing were observed. Participants washed their hands with soap on four percent of these occasions and with water only on 25% of occasions. On 71% of the critical occasions for hand-cleaning no action was taken by study participants. Soap was frequently observed to be used for other tasks like laundry, dishes and bathing. People often splashed their hands with water during their daily tasks rather than taking time to specifically go and wash hands with soap. For example, people often ate at different times, with some people washing their hands before eating and others not. We observed that there was no set place for soap and that people often spent time searching for it, making handwashing inconvenient.

Table 2 summarises presence, wetness and smell data from follow-up visits one and two s during the behaviour trials. The number of towels distributed (n = 123) differs from the overall number of participants (Table 1, n = 128), as children under the age of one were not given their own towel. Relatively few Supertowel were lost or given away during the period of the behaviour trials (n = 10). Most of these losses occurred in one household of female minors. At the time of follow-up visit one 49% of Supertowel were either wet or damp indicating use that day. This figure rose to 60% at follow-up visit two. In 17 households at least one Supertowel was wet or damp at the time of follow-up visit one and by follow-up visit two all 19 households had at least one wet or damp towel. Many of the households said that they felt comfortable sharing the Supertowel between family members and had chosen not to use all the towels each day. Sharing was considered preferable because it allowed families to store some of the Supertowel and ‘save them’ for future use. The majority of Supertowel did not smell at follow-up visit one (68%) or follow-up visit two (80%). Smell challenges reduced over the course of the trials, indicating that participants developed mitigating strategies.

**Table 2:** Statistics related to the use and location of the Supertowels among Eritrean Refugees in Hitsats Camp who participated in the behaviour trials–disaggregated by time of visit.

Follow up visit 1		Follow up visit 2	
**Attrition (n = 123)**		**Attrition (n = 123)**	
Towel present	95 (77%)	Towel present	78 (63%)
Towel out with person	12 (10%)	Towel out with person	25 (20%)
Misplaced / lost/ given away	11 (9%)	Misplaced / lost/ given away	10 (8%)
Missing data	5 (4%)	Missing data	10 (8%
# of households with one or more ST missing	4 (21%)	# of households with one or more ST missing	5 (26%)
**Location (n = 123)**		**Location (n = 123)**	
In house	87 (71%	In house	*62 (51%)*
On person	20 (16%)	On person	*41 (33%)*
Misplaced / lost/ given away	11 (9%)	Misplaced / lost/ given away	*10 (8%)*
Missing data	5 (4%)	Missing data	*10 (8%)*
**Wetness (n = 95)**		**Wetness (n = 78)**	
4: Wet	42 (42%)	4: Wet	43 (56%)
3: Damp	5 (5%)	3: Damp	4 (5%)
2: Almost Dry	12 (12%)	2: Almost Dry	3 (4%)
1: Dry	41 (41%)	1: Dry	28 (35%)
# of households with at least one ST that was wet or damp	17 (90%)	# of households with at least one ST that was wet or damp	19 (100%)
# of households with at least one ST that was dry	13 (68%)	# of households with at least one ST that was dry	11 (58%)
**Smell (n = 95)**		**Smell (n = 78)**	
4: No smell / pleasant odour	64 (68%)	4: No smell / pleasant odour	62 (80%)
3: Slight smell	21 (21%)	3: Slight smell	13 (16%)
2: Smelly	7 (8%	2: Smelly	3 (4%)
1: Very smelly	3 (3%)	1: Very smelly	0

Note: Denominators for wetness and smelliness statistics were based on the number of towels at home during the visit. Denominators for location statistics were based on the number of towels distributed to each household, minus any lost or misplaced towels. ST = Supertowel

Reactions to the product during the initial visit were positive (Table 3). In general, respondents believed the verbal description of the Supertowel provided by the research team. That is to say that they believed the product would effectively remove germs from hands and kill them. They also thought the SuperTowel would be more economical, convenient and water saving than handwashing with soap.

**Table 3:** Perceptions of the Supertowel among Eritrean refugees in Hitsats Camp at the time of the initial visit to households who participated in the behaviour trials.

Belief in the Supertowel ability to kill germs	HH19: *“Using water and soap may clean our hands but won’t kill the pathogens*, *but with the ST as you have told us*, *all those micro-organisms are going to be killed*, *so this feeling is building my confidence*.*”* HH14: *“I am happy that I have it*, *and that I am going to use it for handwashing*, *and that it will save water and kill all the pathogens on my hands at the same time*.*”*
Perceived ability of the Supertowel to save water	HH07: *“Primarily since there is shortage of water in the area it is going to be helpful in saving water while cleaning and killing the pathogenic bacteria on our hands*.*”* HH03: *“With the shortage of water in our camp this product will be very helpful in washing our hands with a little amount of water*.*”*
Perceived ease of using the Supertowel	HH13: *“It would not be easy to carry and use a wet soap whenever we are travelling*, *but with this ST*, *with its pouch*, *it can be easily used*.*”* HH04: *“When I am going outside the house traveling somewhere*, *I am going to take it with me to use it wherever*, *whenever I want to*.*”* HH09: *“No*, *I don’t think there would be any difficulty because it seems easy and quick to use*.*”*
Perceived economic benefits of the Supertowel	HH11: *“The other advantage is that we may not have to buy any soap for handwashing at last*, *which is an economical advantage*.*”* HH13: *“Economically we may not have to worry about buying soap for at least handwashing*.*”*

When we interviewed participants during follow-up visits one and two, people’s experiences of using the Supertowel were largely consistent with their initial impressions and expectations (Table 4). The majority of people reported that the Supertowel had enabled them to wash their hands more frequently and that it left their hands feeling nicer than handwashing with soap. People also reported that they had tried using the Supertowel for other purposes such as keeping themselves cool, face wiping, swatting away flies, cleaning wounds, wiping surfaces and bathing.

**Table 4:** Experiences of Supertowel use as reported by Eritrean refugees in Hitsats Camp who participated in the behaviour trials–data generated during the first and second follow up visits.

Belief in the Supertowel ability to kill germs	HH08: *“When I wash my hands with the ST and look at it*, *it feels clean and makes me feel confident that it is killing and removing all the germs*.*”* HH15: *“We are feeling very happy that the towel is preventing us from germs and the like*, *which makes us confident that we are clean*.*”* HH13: *“Here using the ST is like getting three advantages at the same time; one*, *I am cleaning my hands*, *two it is killing and removing the pathogens from my hands*, *and the third advantage is I get my hands dry easily*.*”*
Ability of the Supertowel to save water	HH11: *“This week it was busy and we had to buy lots of water for the special occasion (baptism)*, *but because the whole family have been using the ST for our hand and face washing we have saved lots of water*.*”* HH03: *“As of my own observation this ST has the ability to save water*, *it didn’t drop any water so it enables me to wash my face with little amount of water*.*”*
Ease of using the Supertowel	HH14: “*All the time*, *all day long here hygienic activities are something that must be done*, *and the ST is easy to access and use frequently*.” HH18: *“We use the ST when we are unable to find soap and water*, *when we are in a hurry like when I go to church*. *I like that it doesn’t cost me time and that I can wash my hands and face while I am traveling*.*”*
Economic benefits of the Supertowel	HH29: *“Economically it is helpful because we don’t have to buy soap for hand washing frequently*.*”* HH16: *“It makes things easy*. *It means that I don’t have to go to the shop to buy soap I just simply wet the towel with water and use it to wipe my kids’ hands and face*.*”*
Ability of the Supertowel to increase handwashing frequency	HH08: *“I have started keeping it nearby me while I am cooking and doing other activities and it did increase the number of times I wash my hands*.*”* HH09: *“When I am busy with domestic activities I used to not bother washing after every minor activity*, *like making injeera while cleaning the house at the same time*, *but now because of the accessibility of the ST I am washing my hands after every one of these minor activities*.*”* HH04: *“Sometimes it is difficult to get soap and we are forced to rinse with only water*, *but with this ST the number of times that I wash my hands in a day has increased a lot*.*”*
Perceived pleasantness of using the Supertowel	HH17: *“In the last ten days I have been using only the ST continuously for handwashing*, *enjoying it*, *experimenting and trying to compare it to handwashing with soap*, *and I really like the feelings it [the ST] left on my hands after I used it*. *Even for face washing it is refreshing and softens far better than using soap and water*.*”* HH02: *“I have used the ST for the last five days*, *I am not sure if it kills all the microorganisms in our hands*, *but it cleans my hands and makes my skin smooth and shining*. *I can attest that the ST is not just a simple towel*, *there must be something you have added to it to clean our hands and make our skin so smooth*.*”*
Use of the Supertowel for purposes other than handwashing.	HH15: “*I am using the ST not only for handwashing but also for cleaning my breast when I am about to breastfeed my baby*.*”* HH11: *“This week [my son] is not well especially in his eyes and we have been wiping out and cleaning his eyes and face with the ST continuously and he seems to like it that we are not washing him with the water and soap*. *I guess it would have been painful for him if it was with soap and water*.*”* HH08: *“Yes I have started to use it for washing and wiping the coffee table and related equipment*.*”*
Times when the Supertowel is particularly useful	HH03: *“Last time there was a shortage of water and it was impossible to wash manually (with water and soap)*, *I wet this ST with a little water to wipe my hands and face*. *I was glad to have it at that time*.*”* HH16: “*Sometimes*, *while I am baking injeera the kids may ask to defecate so I help them with that and wash my hands with the towel which makes things easier…it helps me in saving water and soap and enables me to wash my hands more frequently*.” HH11: “*I liked using it when I had sick eyes [an eye infection]*, *because it does not hurt it*, *rather it feels good on my face*.*”*

Participants identified several barriers and facilitators to the effective use of the Supertowel. By follow-up visit one, it became clear that some of the Supertowel had started to develop a bad smell. There was concern that this could become a barrier to use, however participants still claimed to use the towel, and many had taken steps to mitigate the smell of the towel; either by washing it, leaving it out of the bag (the bag was not treated with the antimicrobial technology), or hanging it to dry:

HH04: *“It did not take us long [to adapt]*, *except for the challenge of the unpleasant smell we faced at first*, *but once we managed it*, *it was easy to adapt and use it frequently*.*”*

HH08: *“It’s obvious that any piece of cloth could smell bad if it is kept in a bag while it is wet*, *therefore we were able to air and get rid of the bad smell*.*”*

Location also emerged as a potential barrier or facilitator to use. Those who kept the towel in an accessible location self-reported more consistent use of the product, than those who did not. For example, one family explained that since follow-up visit one they had changed where they stored their Supertowel and this had facilitated increased use:

HH02: *“Now we all just hang our towels on an accessible place on the wall*, *so we can easily wash our hands with it*.*”*

At the time of follow up visit one, several participants explained that they were struggling to adapt to the new mode of hand-cleaning. Some households explained that they preferred to use the Supertowel after washing their hands with water and / or soap to avoid dirtying it or making it smell:

HH17: *“We did not use it today*. *But there was nothing preventing us from using it*. *We are just getting adapted to it*.*”*

HH14: *“Generally*, *whenever there is any visible dirt on my hands*, *I wash them first with water and then use the ST*.*”*

However, by follow up visit two the majority of households reported fully adjusting to use of the Supertowel for hand-cleaning. Some even reported becoming addicted to it. This suggests that the time required for adopting to this new method of hand cleaning is quite rapid (less than 12 days).

HH04: *‘Previously when I started to use this [the ST]*, *it was after rinsing my hands with water*. *But these days I have developed the culture of washing my hands frequently with only the ST*.*’*

HH17: *‘The towel is always on my hands wherever I go and I think I am getting addicted to using it*.*’*

Households with young children, people with disabilities or dependent older people, explained that the Supertowel was beneficial in providing care. During the observations, for example, one mother whose son was incontinent was observed to only be able to clean him post-defecation. She explained that the Supertowel has helped to keep his face and hands cleaner throughout the day:

HH16: *“[The ST] was very useful*. *He was nagging and causing trouble when he was washed with soap and water*, *but now when he is washed with the ST he is happy and comfortable*.*”*

Another mother with four young children explained how the Supertowel helped her to manage the hygiene of her family:

HH05: *“I can only say that I like it so much…once I dip and wet one towel*, *it is enough for me to clean all of my kids…Most importantly the kids like it so much and are using it frequently*.*”*

At the end of the behaviour trial, only one person indicated that they might not continue using the Supertowel in the future.

Generally, the prototype of the Supertowel that we used in this study was well liked. People were hesitant to make recommendations relating to the design and the look of the product because they felt that its purpose was more important than its physical appearance. However, some participants suggested that more could be done to improve the bag and the uniqueness of the product so that it could be readily differentiated from other microfiber cloths:

HH18: *“For the bag*: *if you could make it smaller and suitable to carry like a hand purse*.*”*

HH15: *“I think the bag is made up of plastic and it doesn’t pass air to the towel*. *So*, *I recommend making it so it could somehow pass air to keep the towel fresh*.*”*

HH18: *“You should do something which makes the ST unique*. *Whether*, *it’s the design or the shape or colour*.*”*

HH15: “*It must have its own label or tag which might be easily identified*.*”*

In focus group discussions, participants identified some of the current barriers to handwashing with soap in the camp. These included the quantity and quality of the soap that is distributed; the irregularity and scarcity of water supply; and the overall living conditions in the camp:

Focus Group Discussion–M: *“The soap is never enough*, *and it is not good quality*.*”*

Focus Group Discussion–M: *“As my friend mentioned*, *we only get one [bar of soap each per month] …and it stops making foam and we are forced to throw it away*.*”*

Focus Group Discussion -F: *“We sometimes could not find water to drink*, *let alone for our hygiene*.*”*

Focus Group Discussion -M: *“We are living in over-crowded rooms*, *like sometimes we have 30 people in a single room*. *This is another reason making it difficult to practice handwashing regularly*.*”*

As people tried the different types of hand-cleaning products, participants generally expressed surprise at how pleasant it was to use the Supertowel:

Focus Group Discussion–F: *“Before we tried it*, *we would have said that using the soap and water is much better than this*. *But once we tried it*, *I am witnessing how good and efficient a product this is*, *in many ways; cleaning our hands*, *killing the germs*, *and saving water all in one*.*”*

Focus Group Discussion -M: *“Remember what I said when I washed my hands with soap*? *There was moisture and some residue from the soap left*, *and smell*. *And when I washed with alcohol rub I told you that even though it was very clean*, *it felt dry and rough because it evaporated all the wetness*. *But now*, *when I clean my hand with the ST*, *every residue and smell has been removed*.*”*

However, there was one female participant who disagreed with other members of her group. She explained: “*I like all the things you [group members] said about the ST but I have not felt like I am clean compared to when I wash with water and soap*.”

The participants were asked to rank each of the hand-cleaning products against different criteria. This data is summarised in Table 5. Both men and women considered the Supertowel to be second to alcohol hand rub in its ability to save water. This makes sense given that alcohol hand rub requires no water at all. In terms of desirability, women ranked the Supertowel second to a bar soap which claimed (on the packing) to be particularly effective in killing germs. Through the discussions, they explained that one of the reasons for this was that bar soap can also be used for dishes and laundry, whereas the Supertowel cannot. Men did not share this concern and ranked the Supertowel the highest in terms of desirability. All other rankings for the Supertowel were homogenous across the two groups. Responses also indicated that the soap which is currently distributed by UNHCR rates poorly against many of the criteria.

**Table 5:** Rankings of hand cleaning products from Eritrean refugees in Hitsats Camp who participated in the focus group discussion.

	Desirability		Perceived Cost		‘Long Lastingness’		Perceived Ability to Save Water		Perceived Effectiveness at Killing Germs	
	Men	Women	Men	Women	Men	Women	Men	Women	Men	Women
**Rankings** (highest ranked at the top)	**4**	5	**4**	**4**	**4**	**4**	3	3	**4**	**4**
	2	**4**	3	3	5	1	**4**	**4**	3	3
	3	2	2	2	6	2	1	2	2	2
	5	1	5	5	1	5	6	1	5	5
	6	3	6	6	3	6	5	5	1	6
	1	6	1	1	2	3	2	6	6	1

1 = UNHCR Soap, 2 = Liquid Soap, 3 = Alcohol Hand Rub, **4 = Supertowel**, 5 = Bar Soap (with health benefits emphasised), 6 = Bar Soap (with beauty benefits emphasised)

**Fig 1:**
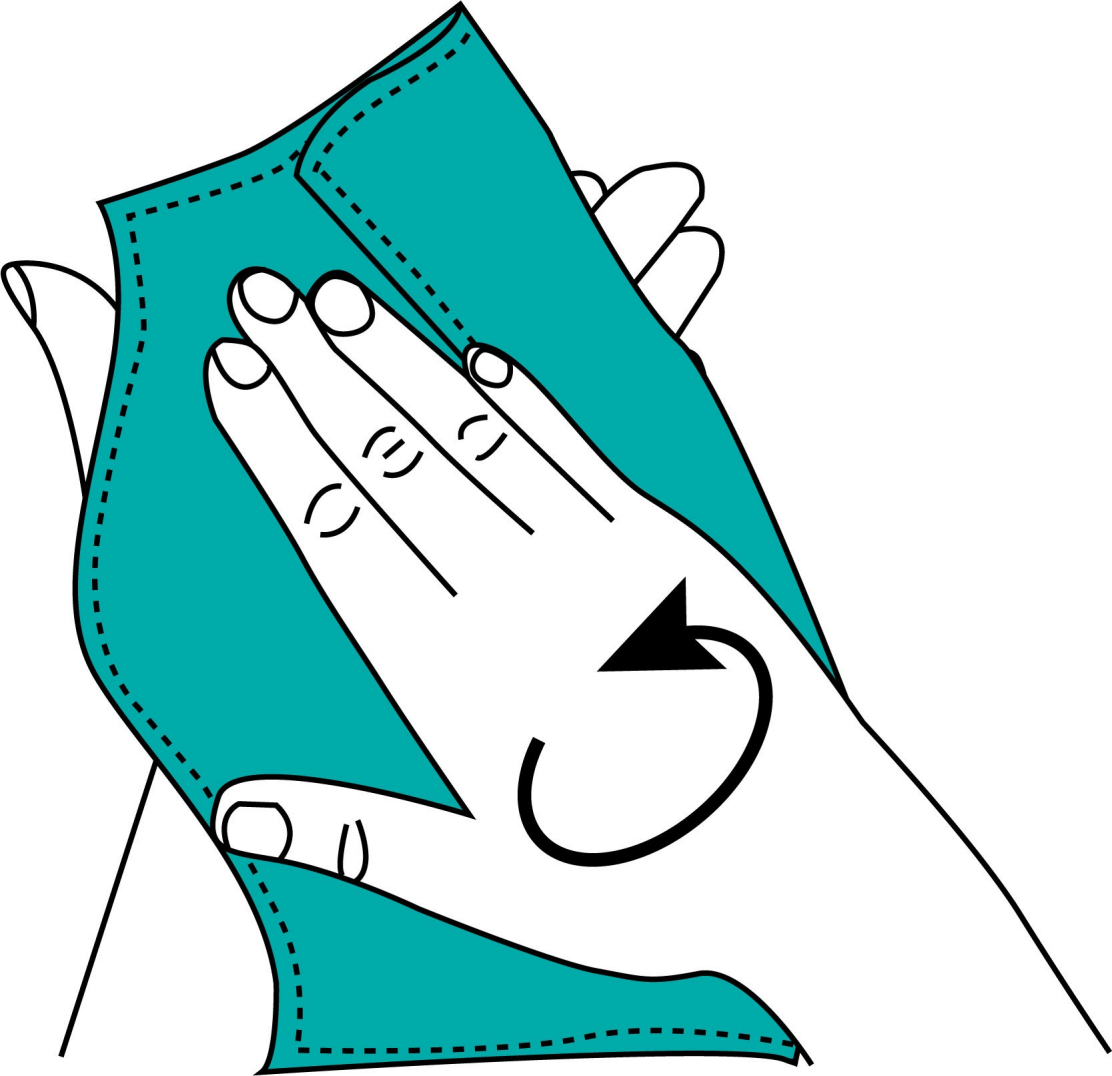
Initial image used in the focus group discussion.

**Fig 2:**
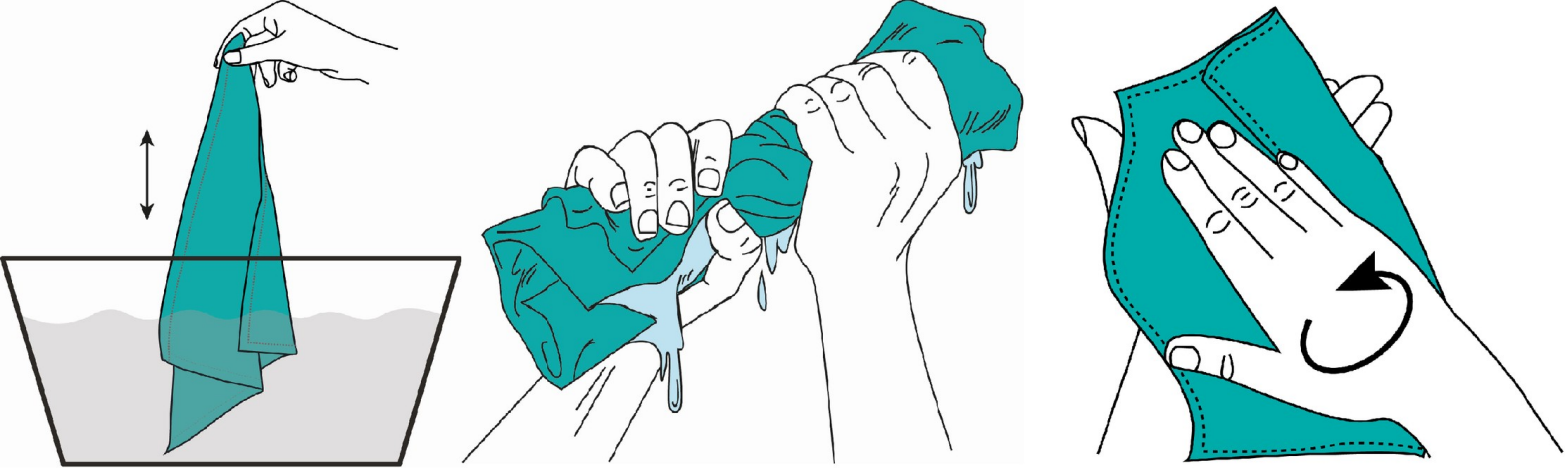
Second set of images used in the focus group discussion.

During the final focus group discussion with women, the intuitiveness of the Supertowel was tested by introducing the product without instructions and asking for feedback on how the women would use the product. Without any instructions, the women initially felt that the product could be used after for drying themselves or their children after bathing or that it could be used for cleaning up around the house (wiping coffee tables or cups). None of the respondents initially guessed the product was for hand-cleaning. When presented with Fig 1 women guessed that the product was for hand drying but not hand-cleaning. When women were presented with Fig 2 they were able to use the Supertowel as intended for hand-cleaning. The instructions show that in order to use the Supertowel it must first be dipped into water to soak the towel thoroughly, then rung-out, and then wiped over the hands to clean them. Even once the women had understood the instructions, they remained unconvinced that the product could clean their hands as well as soap and water.

Women in this final focus group discussion recommended instead that distribution of the towels should occur with some in-person explanation of how the Supertowel works and what makes it efficacious in order for it to be accepted. Specifically, they explained that it would be good to have it explained by local ambassadors (citing themselves as examples) who could draw on their own experience with the product:

Focus Group Discussion -W: *“It would be excellent if we all have it and are using it*, *if we could teach others too based on our real experience*.*”*

Some responses from the behaviour trials also indicated that this interpersonal communication is likely to be key for establishing trust in the product:

HH16: ‘*For us it was the experience that made us believe that it actually did kill the germs*. *But for others*, *as they didn’t have a chance to try it*, *I wouldn’t quite be sure that they would believe in the ST’s ability to kill germs*.*’*

HH06: *“If you are going to distribute the ST*, *it would be good if you first teach every house on how to use it*, *and how valuable it is*.*”*

## Discussion

Baseline observations and discussions with participants confirmed our assumptions about the challenges of practicing handwashing with soap in a camp context. Not only did people report having insufficient access to water and soap, but their inability to practice handwashing was compounded by condensed living environments, a lack of social support for handwashing and an absence of handwashing facilities to cue handwashing behaviour or make it more convenient. Studies of handwashing behaviour in other locations have identified similar barriers [19, 22, 24–26] The Supertowel was generally well liked and seen as a product which could adequately meet hygiene needs in humanitarian contexts. Participants in the behaviour trials trusted the efficacy of the product from the outset and were able to recognise its potential benefits, including that it would save water and the amount of money spent on soap. As the behaviour trials continued, participants reported that their initial impressions of the product were consistent with their actual experience of using it. They also reported increased frequencies of handwashing associated with the ease of using the Supertowel. Wetness rankings supported this by suggesting that 19 out of 19 (100%) households had at least one Supertowel in use (that was wet or damp) at the time of follow-up visit two. Findings from the focus group discussions confirmed the acceptability seen in the behaviour trials. Both men and women ranked the Supertowel highly against all criteria and substantially better than the soap that was being distributed by camp authorities. Adaption to this new mode of hand-cleaning appeared to occur quickly, with some individuals stating they felt ‘addicted’ to the towel just 12 days after the initial distribution. Many participants reported using the SuperTowel for reasons other than hand cleaning, but that this did not deter them from subsequently using it on their hands. Alternative uses included cleaning surfaces and dishes, face cleaning, and bathing.

This study also identified several ways in which the product could be improved to make it more suitable for crisis-affected populations. Firstly, the carry bag was liked by many but the current design still appears to be imperfect; several behavioural trial participants decided not to use it at all and some people felt it was too large and it made the Supertowel smell unpleasant. Secondly, the study highlighted the need to better understand potential mechanisms for distributing the product and the need to increase the intuitiveness and perceived authenticity of the Supertowel. Results from both the focus group discussions and behaviour trials suggested distribution of the product should be complemented with some kind of interpersonal communication which explains how the Supertowel works and how it is to be used and maintained. Distribution en masse though hygiene kits may be insufficient to guarantee use. This interpersonal package would need to bring together technical data on the product (developed through further laboratory tests mimicking real-use conditions) and design elements to communicate the content in an acceptable manner. Since the conclusion of the field work we have been able to modify the bag and the Supertowel to reduce all odour (even if left wet in the bag for 144 hours) and we are planning further research to explore distribution mechanisms.

Every attempt was made to mitigate potential biases during the study, including using wetness of the Supertowel as a proxy indicator for use, and triangulating data from a range of qualitative methods to determine acceptability. Blinding participants during behaviour trials was not possible, and therefore we were unable to use observation as a means of assessing hand-cleaning behaviours with the Supertowel. Self-reported hand-cleaning behaviour (as was generated through the behaviour trials) may have led to overestimates of actual practice, as shown in other studies [27–29]. Although we specifically asked participants to critique the product, there is also a risk that participants underreported what they did not like about the Supertowel. This social desirability bias may have been worsened by the fact that some of the research team were foreigners and others worked for the Danish Refugee Council, which had been providing services in the camp for several years. Due to time constraints, this study was conducted during the rainy season in Ethiopia, when households generally have access to higher quantities of water. Increases in water quantity typically result in households being able to allocate more water to hygiene-related tasks [30, 31]. Even though the Supertowel uses substantially less water than handwashing with soap it is plausible that use of the product may decrease in the dry season. This is unlikely to be a major concern given that in Hitsats camp, in the wet season, people’s water access is only marginally above the minimum basic water requirements per person per day [32]. During the period of the behavioural trials, participants also reported water outages lasting for a whole day and indicated that it was at these times when the Supertowel was most beneficial.

Historically, products distributed to crisis-affected populations have not been designed with their inputs or ideas. This research contributes to challenging this norm by including participant feedback as part of an iterative design process. Within the water, sanitation and hygiene sector there are now a number of projects in humanitarian contexts which aim to foreground the voices of crisis-affected populations and involve them in design [33–35].

## Conclusion

By triangulating data from a range of qualitative methods we were able to demonstrate that the Supertowel is an acceptable product and a feasible alternative to soap in contexts where handwashing is challenging. Use of the Supertowel was high (90% of households at follow-up visit one, and 100% of households at follow-up visit two), and self-reported adoption to the new behaviour occurred quickly. This pilot study demonstrated the value of engaging crisis-affected populations in the design of products which may be used by them in the future. Specifically, this process identified required modifications to the Supertowel design, such as improving the carry bag and addressing smell issues. The study also identified areas of future research including the need to compare different distribution models and to evaluate the use of the Supertowel at scale, over a longer period of time, and using observed measures for hand-cleaning.

## Supporting information

S1 Guide Behaviour trial guides used in this study. (DOCX)

S2 Guide Focus group discussion guides used in this study. (DOCX)

S1 Table The COREQ checklist with responses relating to the design and implementation of this study. (DOCX)
