# Supplementary material for: Could the Supertowel be used as an alternative hand cleaning product for emergencies? An acceptability and feasibility study in a refugee camp in Ethiopia
Source: PLoS One. 2019 May 6;14(5):e0216237. doi: 10.1371/journal.pone.0216237 (PMC6502319; doi:10.1371/journal.pone.0216237)
Supplement: S1 Guide — (DOCX) [file pone.0216237.s001.docx]

# Behaviour Trials

## Overall Process:

1. The consent process for this method must be done with the whole family. For the behaviour trials potential participants must receive information about what is being asked of them but also information about the Super Towel product itself. Potential participants will receive information verbally (and in written form) about the Super Towel but will be given the chance to interact with the product and use the product for hand cleaning (with guidance from the research team) prior to agreeing to participate. Consent and assent will then be provided in the same manner as for observation.
2. Researchers will give the household one Super Towel (one of the 3 prototypes) per family member. This includes providing a Super Towel for all infants and young children (although this will be given to their parents. They will be given the information sheet about the Super Towel to keep as well as a guide about the four critical times for hand cleaning which the research team will talk them through.
3. At the point of initiation, a short questionnaire about current handwashing practices will be completed. These will be directed to the female household head. The research team will then ask several more open-ended questions to the female household head about their initial attitudes to the Super Towel and their expectations about how the behaviour trial will go. The questionnaire and the interview guides are provided at the end of these instructions.
4. After the initial discussion we will arrange a follow up time with each family. The first follow up will be on days 4-6 of their trial and the second follow up will be on days 9-11. The slight variation in days is due to the anticipated logistical limitations of trying to reach all families in a similar time period. At each of these time periods we will take time to speak with the female household head and other family members if they are available. At the second follow up we will conduct a semi-structured interview (guide below) and at the final interview will conduct another semi-structured interview.

## Initial interview prior to starting the behaviour trial

- Now that you have seen and used the Super Towel, do you think your family will encounter any challenges using it over the next 10 days?
- What is appealing to you about the Super Towel?
- What is unappealing to you about the Super Towel?
- Do you think it will make hand cleaning any easier for your family? Why?

## Process for first follow up visit

**Step 1: Rapid observation**

- How many STs are visibly present upon arrival to the household
- Where are they (e.g. on a person vs stored somewhere)

**Step 2: Questionnaire**

Conduct socio-demographic questionnaire for all members of the family

**Step 3: Show us the SuperTowels**

Get each person to bring out their SuperTowel.

- Is everyone in the house able to find theirs?
- If some are missing find out what happened?

Get each person to show the SuperTowel?

- How many are damp?
- How do they smell?

**Step 4: Interview**

Interview guide:

- What have your experiences been using the Super Towel so far?
- What have you enjoyed about cleaning your hands with the Super Towel?
- What have you disliked about cleaning your hands with the Super Towel?
- Have you and your family members encountered any challenges when using the Super Towel before eating? What were these? Can you think of any ideas for overcoming these challenges?
  - In Ethiopia it can be a mark of respect to pour water for others as they wash their hands. With the Super Towel you can’t do that. Is that something that you miss?
- Have you and your family members encountered any challenges when using the Super Towel after going to the toilet? What were these? Can you think of any ideas for overcoming these challenges?
- At what other times during the day have you used the Super Towel for handwashing?
- Have you tried using the SuperTowel with water that is not clean? How did you feel about this?
- Have you encountered any instances when it is inconvenient to clean your hands with the Super Towel?
- When you use the Super Towel do your hands feel clean? Do they feel as clean as when washed with soap?
- Do you feel confident that all the germs are being killed – why/why not?
- Did you end up getting your SuperTowel’s mixed up? Did this bother you to share a SuperTowel with someone else?
- Has the Super Towel been able to be used by all members of the family including those who are very young, very old or have a disability?
- Have your neighbours noticed you using the product? If so what did they ask and what did you tell them about the product?
- Have you found that the Super Towel is useful for any other purposes around the home?
- Have you found that you have had to do anything to maintain the SuperTowel? (e.g. airing it, washing it, etc)
- Has your SuperTowel started to smell? How did you feel about this? Did it make you want to use it less?
- Are there any changes you would recommend to improve the product?
- Would you recommend it to others?
- Do you think visually appealing? Is there anything we could do to make it more beautiful?

## Process for the second follow up visit

**Step 1: Rapid observation**

- How many STs are visibly present upon arrival to the household
- Where are they (e.g. on a person vs stored somewhere)

**Step 2: Show us the SuperTowels**

Get each person to bring out their SuperTowel.

- Is everyone in the house able to find theirs?
- If some are missing find out what happened?

Get each person to show the SuperTowel?

- How many are damp?
- How do they smell?

**Step 3: Interview**

Interview guide:

- Have your experiences using the supertowel changed in any way since we last spoke?
- Last time we spoke we set several challenges for you, were you about to try these?
  - Use grey water
  - Rinse and hang up the towel daily
  - Try to only use the towel when leaving the toilet
  - Take the towel with you when you leave the house
  - Hang some of the towels in places which were easy to access/ cue use
- Have you and your family tried using the SuperTowel in any other different ways since last time we spoke?
- Have you noticed that the SuperTowels started to smell? How did you deal with this?
- Have you found that the Super Towel is useful for any other purposes around the home?
- Can you tell us about one particular occasion in the last 10 days when you really appreciated having the SuperTowel.
- Are there any changes you would recommend to improve the product?
- We have noticed that not everyone is using the bag – do you find the bag convenient or is it unnecessary?
- Is this a product that you would like to keep using in the future?
- Do they foresee any long-term obstacles in using or maintaining the Super Towel?
- How would they convince others in their community that it is a product worth using?
- Ideally we would hope that this product could be distributed for free to refugees like you, however if people were to pay for the Super Towel how much do you think would be a reasonable price?
- When we first spoke with you we asked you what is appealing to you about the Super Towel, after using it for 10 days what appeals to you now about the product?
- We also asked you what is unappealing to you about the Super Towel, after using it for 10 days what do you find to be unappealing about the product now?
- Overall do you think that the Super Towel makes hand cleaning any easier for your family? Why?
- Overall do you think that the Super Towel requires less water than handwashing with soap?
