# Supplementary material for: Could the Supertowel be used as an alternative hand cleaning product for emergencies? An acceptability and feasibility study in a refugee camp in Ethiopia
Source: PLoS One. 2019 May 6;14(5):e0216237. doi: 10.1371/journal.pone.0216237 (PMC6502319; doi:10.1371/journal.pone.0216237)
Supplement: S2 Guide — (DOCX) [file pone.0216237.s002.docx]

## Focus Group Discussion – version 1

Process:

1. Potential participants should be approached individually, informed about the FGD and the consent process undertaken prior to them arriving at the FGD.
2. For the FGD there should be one research assistant who acts as the primary facilitator of the discussion and another research assistant who is the scribe. The scribe should capture the key things that everyone says.
3. To start, get participants to introduce each other and say their favourite food, colour or a particular skill they have. These are all fairly neutral questions and are preferable over more personal topics given the experiences people may have recently been through.
4. Then explain the rules of the focus group these should include the following:
   1. There are no right or wrong answers to anything we discuss today and it is important that we respect the opinions of others in the group.
   2. In this discussion we are interested in hearing everyone’s opinion. So we will encourage you all to take turs speaking and you should try not to talk over or interrupt other people while they are speaking.
   3. We want you to feel comfortable sharing your opinion with us and so we would ask that all of you agree to not share what we discuss here with those outside the group after we finish.
5. Start by asking the participants whether there are any factors that make it challenging for them and their families to wash their hands with soap. Even if they personally find it easy get them to think about factors that might make it difficult for some other people in the camp.
6. Introduce participants to 5 different hand cleaning products (ideally bar soap, liquid soap, laundry soap, alcohol hand rub, and the super towel). Provide a basic explanation of how each product should be used and how it works. Ask participants to list the good and bad things about each product.
7. Set up some hand cleaning stations and get people to try each of the products (in different orders) as each person tries each product get them to think about what they like and don’t like, how their hands feel and how their hands smell after using the product.
8. Bring all the products back to the centre of the room. Explain that you would like participants to rank the products according to different criteria.

The scale parameters we will assess include:

- 1. Undesirable – desirable
  2. Unhygienic - Hygienic
  3. Unpleasant – pleasurable
  4. Consumed quickly - Long-lasting
  5. Strange – familiar
  6. Something that I really wouldn’t want to use – something that I really would want to use
  7. Something that the village chief is not likely to use – something that the village chief is likely to use
  8. Not very effective in killing germs - Very effective in killing germs
  9. Hard to use – easy to use
  10. Water wasting – water saving.

1. At the end of the FGD thank the participants for their time, explain how the information they shared will help us improve the product and see if they have any further questions.

## Focus Group Discussion – version 2

Process:

1. Potential participants should be approached individually, informed about the FGD and the consent process undertaken prior to them arriving at the FGD.
2. For the FGD there should be one research assistant who acts as the primary facilitator of the discussion and another research assistant who is the scribe. The scribe should capture the key things that everyone says.
3. To start, get participants to introduce each other and say their favourite food, colour or a particular skill they have. These are all fairly neutral questions and are preferable over more personal topics given the experiences people may have recently been through.
4. Then explain the rules of the focus group these should include the following:
   1. There are no right or wrong answers to anything we discuss today and it is important that we respect the opinions of others in the group.
   2. In this discussion we are interested in hearing everyone’s opinion. So we will encourage you all to take turs speaking and you should try not to talk over or interrupt other people while they are speaking.
   3. We want you to feel comfortable sharing your opinion with us and so we would ask that all of you agree to not share what we discuss here with those outside the group after we finish.
5. Show the participants the Towel (do not call it the SuperTowel). Ask them what they could use this product for.
6. Present image 4. Explain that if this image came with the product, how would they use it? Get them to demonstrate
7. Present image 2 together with image 4. Ask participants how they would use the product if these two images came with the product. Get them to demonstrate.
8. Present images 1-4 in order. Ask them if this helps them understand the process more clearly.
9. Ask participants if they would trust a product like this for hand cleaning. Do they think it would remove and kill germs as effectively as handwashing with soap?
10. Show the participants the ‘under the microscope images’ ask them what they think they see. Do these images help convince them that the product effectively kills germs or would they still want more information? What kind of information?
11. Can the participants identify other benefits of this product? Get them to list these. If they are not mentioned probe on the following topics: water use, ease of access, re-usability.
