# Supplementary material for: Could the Supertowel be used as an alternative hand cleaning product for emergencies? An acceptability and feasibility study in a refugee camp in Ethiopia
Source: PLoS One. 2019 May 6;14(5):e0216237. doi: 10.1371/journal.pone.0216237 (PMC6502319; doi:10.1371/journal.pone.0216237)
Supplement: S1 Table — (DOCX) [file pone.0216237.s003.docx]

Table 1: Description of the COREQ criteria as they apply to this research

| Personal Characteristics | | |
| --- | --- | --- |
| 1 | Interviewer/facilitator | Interviews were led by SW with KD providing simultaneous translation for both questions and responses. THL was present for all interviews, providing additional prompts to the interview team as necessary. |
| 2 | Credentials | SW is a PhD candidate in public health and behaviour change, she holds an MSc in Public Health in Developing Countries and a BA in Society and Culture. KD has a BA in Applied Biology. THL holds a Msc in Chemical Engineering. JP has an MSc in Public Health in Developing Countries and a BA in Humanities. |
| 3 | Occupation | SW is a Research Fellow at the London School of Hygiene and Tropical Medicine (LSHTM). At the time of the study JP was working as a Research Assistant at LSHTM. At the time of the study KD was working as a consultant translator for the Danish Refugee Council. THL developed the SuperTowel and is the Director of Real Relief a humanitarian innovation company. |
| 4 | Gender | SW and JP are female. KD and THL are male. We also worked with male and female social workers in the refugee camp during the data collection. |
| 5 | Experience and training | SW has conducted qualitative research in Indonesia, Nigeria, DR Congo, Iraq, Malawi, Papua New Guinea and other parts of Ethiopia. JP has conducted qualitative research in Madagascar, Haiti and Iraq. KD is an experienced translator having worked with refugee populations in this region for several years. THL has developed a range of humanitarian innovations for crisis contexts in the Middle East, Africa and Asia. |
| Relationships with participants | | |
| 6 | Relationship established | We worked with Administration for Refugee & Returnee Affairs at a national, regional and camp level to explain the rationale behind the study. All our work in the camp was supported by experienced staff from the Danish Refugee Council, who had worked in the camp for several years, and Social Workers (camp residents) who knew the population well. The research team had no prior relationships with participants. |
| 7 | Participant knowledge of the interviewer | Prior to recruitment we took time to clearly inform participants about the study through the voluntary informed consent process. This typically took about 15-20 minutes per household. This process clearly explained the goals of the research. |
| 8 | Interviewer characteristics | The composition of our research team (with foreign researchers, product develops, local NGO staff and camp residents) meant that we brought diverse perspectives and different biases to the research. We endeavoured to balance these subjectivities by taking an hour at the end of each day to reflect on the findings and our perspectives. THL provided a supportive role throughout the data collection but because he had a vested interest in the product he was not involved in the data analysis. |
| Study design | | |
| 9 | Methodological orientation and theory | This research was not firmly grounded in one theory but was influenced by Participatory Action Research (1) and behavioural theories such as Behaviour Centred Design (2) and IBM-WASH (3). |
| 10 | Sampling | Participants were sampled purposively. Social workers from the camp helped the research team to select participants. Participants were selected to include different regions of the camp and to reflect different ages, literacy levels, durations of displacement and religions. |
| 11 | Method of approach | We visited households directly. We explained the purpose of the research, the methods we were asking them to participate in, and provided an overview of the product. |
| 12 | Sample size | 19 households (128 individuals) were part of the behaviour trials. 21 people participated in the focus group discussions. |
| 13 | Non-Participation | No one declined to participate in the study nor dropped out part way through. |
| 14 | Setting of data collection | Observations and behaviour trials and the interviews associated with the behaviour trials were conducted at a household level. Focus group discussions were conducted in social spaces managed by the Danish Refugee Council. |
| 15 | Presence of non-participants | Non-participants were not present for the research. In cases where neighbours visited during the research or people arrived at the social spaces during the focus group discussions, one of the research team would explain to the visitor what we were doing and politely ask them to leave. |
| 16 | Description of sample | A full description of the sample is provided in table 1 in the manuscript. |
| 17 | Interview guide | An interview guide was developed by SW. The guide was different for each of the three visits during the behaviour trials. The guides have been included as part of the supplementary material. Focus group discussion guides were developed by SW and are included as part of the supplementary material. Interview and focus group discussion guides were reviewed by the research team and social workers and refined. |
| 18 | Repeat interviews | Participants in the behaviour trials participated in three sequential interviews about their experience using the SuperTowel. |
| 19 | Audio/visual recording | All interviews and focus groups were audio recorded. |
| 20 | Field notes | Detailed field notes were kept by SW. These captured general reflections of the data collection that were not captured during formal notetaking. The notes also documented the discussions held with the research team at the end of each day. |
| 21 | Duration | The observations took place over the course of three hours. The behaviour trials tool place over 12 days. The initial interview took only about 10 minutes, the first follow up interview took approximately half an hour and the second follow up interview too about twenty minutes. Focus group discussions took approximately an hour and a half. |
| 22 | Data saturation | Data saturation is discussed in the manuscript. Saturation was reached relatively quickly in this study, perhaps because the population had very similar living conditions and were relatively homogenous. |
| 23 | Transcripts returned | All qualitative data was directly translated from Tigrinya to English during the transcription process so transcriptions were not shown to participants. However we have been able to share the research results back to participants through our ongoing connection with the Social Workers in the camp. |
| Analysis and findings | | |
| 24 | Number of coders | JP led the coding and analysis of the data. SW cross-checked a portion of the coding and guided the coding tree development. |
| 25 | Description of coding tree | The thematic areas of the interview and focus group discussion question guides formed the initial coding structure, with additional themes being added as they emerged. |
| 26 | Deviation of themes | Themes were primarily determined a priori based on the interview and focus group discussion question guides. |
| 27 | Software | NVivo 11 was used to aid the data analysis. |
| 28 | Participants checking | A sub-sample of participants were given a lay description of the results and asked if this reflected their experiences. No changes were recommended. |
| 29 | Quotations presented | Quotes from interviews and focus group discussions are used throughout the manuscript and identified by household ID or focus group ID. |
| 30 | Data and findings consistent | Findings are described in the results section and then interpreted in the discussion. |
| 31 | Clarity of major themes | We clearly describe how people’s attitudes and use of the SuperTowel change across time and describe key themes such as perceived pathogenic efficacy, water saving ability, ease of use, economic benefits, ability to increase handwashing frequency, pleasantness of use, adaption and multi-functionality. |
| 32 | Clarity of minor themes | We highlight several examples of where opinions differed. For example one woman in the focus group discussion who graded the SuperTowel much lower than others and a mix of opinions related to the bag. |

1. Baum F, MacDougall C, Smith D. Participatory action research. Journal of epidemiology and community health. 2006;60(10):854-7.

2. Curtis V, Aunger R. Behaviour Centred Design: Towards an applied science of behaviour change. In Press. 2016.

3. Dreibelbis R, Winch PJ, Leontsini E, Hulland KR, Ram PK, Unicomb L, et al. The Integrated Behavioural Model for Water, Sanitation, and Hygiene: a systematic review of behavioural models and a framework for designing and evaluating behaviour change interventions in infrastructure-restricted settings. BMC public health. 2013;13:1015.
